# Role of Endoscopic Ultrasound (EUS) in the Era of Precision Medicine for Pancreatic Cancer Through Next-Generation Sequencing Technology

**DOI:** 10.3390/ijms26178444

**Published:** 2025-08-30

**Authors:** Giulia Peserico, Caterina Stornello, Martina Tessari, Antonio Scapinello, Matteo Curtarello, Mario Gruppo, Ottavia De Simoni, Alberto Fantin

**Affiliations:** 1 Gastroenterology Unit, Veneto Institute of Oncology IOV-IRCCS, 35128 Padova, Italy; giulia.peserico@iov.veneto.it (G.P.);; 2 Division of Gastroenterology, Department of Surgical, Oncological and Gastroenterological Sciences, Azienda Ospedaliera–Università di Padova, 35128 Padova, Italy; martina.tessari@aulss8.veneto.it; 3 Anatomy and Histology Unit, Veneto Institute of Oncology IOV-IRCCS, 35128 Padova, Italy; antonio.scapinello@iov.veneto.it; 4 Immunology and Molecular Oncology Diagnostics Unit, Veneto Institute of Oncology IOV-IRCCS, 35128 Padova, Italy; matteo.curtarello@iov.veneto.it; 5 Surgical Oncology of Digestive Tract Unit, Veneto Institute of Oncology IOV-IRCCS, 35128 Padova, Italy; mario.gruppo@iov.veneto.it (M.G.); ottavia.desimoni@iov.veneto.it (O.D.S.)

**Keywords:** pancreatic cancer, target therapy, genetic mutations, NGS, EUS-FNB

## Abstract

Pancreatic ductal adenocarcinoma (PDAC) is a lethal disease with a dismal prognosis; this is in part due to its late diagnosis at advanced stages. For many patients, medical treatment is the only practicable therapy. In recent years, the development of new technologies that investigate genomic biomarkers has improved the concept of precision medicine to treat patients with PDAC. Through endoscopic ultrasound–tissue acquisition (EUS-TA), tissue from pancreatic cancers can be collected; thus, it has the potential to advance personalized treatment by allowing the assessment of genomic alterations. In this review, we explore the role of EUS in genomic profiling and its strengths and pitfalls in obtaining samples for next-generation sequencing (NGS).

## 1. Introduction

Pancreatic cancer is known for its genetic complexity, and the success of personalized treatment approaches relies heavily on identifying specific molecular alterations within the tumor. Endoscopic ultrasound (EUS)-guided tissue sampling has emerged as a crucial tool in this endeavor, as it allows for the precise extraction of tissue samples from pancreatic lesions, enabling comprehensive molecular profiling. This profiling, in turn, helps identify potential therapeutic targets that can be exploited to design more effective and tailored treatment strategies for patients. Although it is a relatively rare disease, its incidence continues to increase by about 1% annually in both men and women and 1.3% per year. PDAC is the third leading cause of cancer death in men and women, for which mortality has increased in men, from 12.1 (per 100,000 men) in 2000 to 12.7 per 100,000 men in 2020, but has remained stable in women at 9.3–9.6 per 100,000 women [1]. PDAC is estimated to become the second leading cause of cancer-related death by 2030 [2]. The pathogenesis of PDAC is complex and multifactorial; however, modifiable and non-modifiable risk factors have been identified. The former include tobacco usage, alcohol, obesity, dietary factors, and exposure to toxic substances [3].

Non-modifiable risk factors include age, diabetes mellitus, family history of pancreatic cancer, genetics, chronic infections, non-O blood group, and chronic pancreatitis [4]. In particular, genetic factors play a very important role in the etiology of PDAC] [5], and genome-wide association studies (GWASs) have identified some frequent genetic variants associated with pancreatic cancer risk; however, the identified germline genetic alterations explain only 4.1% of the total phenotypic variance of pancreatic cancer [6].

## 2. The Mutational Landscape of Pancreatic Cancer

Familiar pancreatic cancer (FPC), defined as two first-degree-relatives with a family history of PDAC, is estimated to comprise about 4–10% of cases. First-degree relatives of individuals with FPC have a nine-fold increased risk of pancreatic cancer over the general population, and this risk doubles when at least two first-degree relatives in the family have pancreatic cancer, and rises to 32-fold higher in persons with three or more first-degree relatives with PDAC [7]. Moreover, genetic variations or mutations (germline mutations) play a fundamental role in increasing the risk of cancer. Germline pathogenic variants have been identified in several genes involved in the hereditary form of PDAC, such as *BRCA1/2*, *PALB2*, *ATM*, *CDKN2A*, *APC*, *MLH1*, *MSH2*, *MSH6*, *PMS2*, *PRSS1*, and *STK11*. Indeed, some hereditary syndromes also are known to be associated with an increased risk of pancreatic cancer, mainly Peutz-Jeghers syndrome, melanoma–pancreatic cancer syndrome, familial atypical multiple-mole melanoma (FAMMM), hereditary breast–ovarian cancer (HBOC), Lynch syndrome (LS), and familial adenomatous polyposis (FAP). An increased risk of PDAC is present in patients with hereditary pancreatitis or cystic fibrosis [8,9,10]. On this basis, guidelines recommend to consider germline testing in all patients diagnosed with pancreatic cancer to select platinum-based chemotherapy and to select relatives to be screened [11].

Apart from hereditary forms, somatic mutations are far more frequent in PDAC. The genetic landscape of PDAC is characterized by the presence of four frequently mutated genes, represented by *KRAS* (90%), *CDKN2A* (90%), *TP53* (70%), and *SMAD4* (55%) [12,13,14,15,16].

The transcription of the mutant *KRAS* gene determines the production of an abnormal, constitutively activated RAS protein, and this determines an uncontrolled activation of proliferation and survival pathways [17]. The frequent inactivation of the *CDKN2A* gene results in a loss of p16 protein, a negative regulator of the G1-to-S transition of the cell cycle, with the consequent stimulation of the proliferative activity. *TP53* inactivation enables the cells to bypass important control checkpoints at the level of DNA damage and apoptotic signaling. Finally, the frequent loss of *SMAD4* gene results in aberrant signaling by TGF-β [18].

In this context, in dealing with a lethal disease, often diagnosed in advanced stages that require chemotherapy, there is an urgent need to find biomarkers that could help in tailoring the treatment. In fact, knowing the molecular signature of each tumor may contribute to a more fruitful strategy and better outcomes, thanks to personalized therapy. Recently, numerous attempts have been made in this sense, and next-generation sequencing (NGS) technology is a method that enables the simultaneous sequencing of millions of fragments of DNA, with a single test analyzing several genes or gene regions [19]. The main aim is to stratify subclasses of patients with prognostic relevance and possible molecular based-treatment opportunity [20].

Still, the real-world implication of molecular classification remains to be established, and numerous clinical trials are under development to enforce precision medicine in clinical practice. In this field, different initiatives are active all over the world. In the United States, The Pancreatic Cancer Action Network (PanCAN) is conducting a precision medicine program based on personalized treatment options on molecular tumors, called “Know Your Tumor” [21]. In the United Kingdom, a similar initiative was conducted by “Precision-Panc”, in which every registered patient receives a genomic profile of the molecular phenotype of the tumor based on NGS and RNA sequencing. Similarly, in Canada, the EPICC (Enhanced Pancreatic Cancer Profiling) was created [22]. In the first prospective study integrating PDAC subtypes and chemotherapy response (COMPASS trial), the most common genetic alterations were found for *KRAS* (85%), *TP53* (85%), *CDKN2A* (75%), and *SMAD*4 (43%), but an additional 20 actionable mutations were also found comprising *ARID1A* (8%), *BRAF* (2%), *CDK4/6* (7%), *PIK3CA* (7%), *PTEN* (5%), *RNF43* (3%), *BRCA 1* (2%), and *BRCA 2* (4%). Genomic results were available for almost all patients (98%) at a median of 35 days [23,24]. PDAC is characterized by high inter- and intra-tumoral genetic heterogeneity that results in different clinical behaviors among patients. Several molecular profiling studies have demonstrated that molecular alterations in pancreatic cancer are in the range of 12–25%. In a study by Pishvaian et al., with 26% of 1856 patients with PDAC, actionable molecular alterations were identified, and 46 patients received matches therapies. The medial overall survival was 1 year longer than those with actionable alterations receiving unmatched therapy [21]. In the setting of neoadjuvant therapy, a recent phase II trial was designed using validated immunohistochemistry antibodies. Endoscopic ultrasound cell block biopsy specimens from localized PDAC were scored for the expression of six biomarkers, and patients received 8 weeks of profile-directed cytotoxic therapy followed by chemoradiation before surgery. Most patients completed the therapy with an overall survival of 33–41 months [25]. The Individualized Molecular Pancreatic Cancer Therapy (IMPaCT) Trial was the first trial demonstrating the timeliness and feasibility of a molecular-based therapy dividing patients on HER-2 positivity, DNA damage repair alterations, *KRAS* wild-type, or no alterations on NGS [26]. Table 1 summarize major studies.

## 3. The Role of Endoscopic Ultrasound Guided Sampling in Precision Medicine

Despite growing evidence in support of the development of target therapy and the urgent need to define biomarkers predictive of clinical response, significant barriers exist. In fact, the turnaround times for whole-genome sequencing (6–12 weeks), its high cost and poor availability, and the necessity to obtain enough tissue for molecular profiling represent important issues [27].

Finally, many studies have used surgical samples to obtain genomic material to maximize tumor DNA yields, and the lack of adequate tissue specimens in patients with unresectable PDAC has delayed the advent of target therapy in these patients. Furthermore, these reports may not reflect the complete spectrum of known target genomic alterations in PDAC that are potentially found clinically [28].

Since the 1990s, endoscopic ultrasound (EUS)-guided sampling has been used to facilitate the tissue collection of malignant tumors [29]. The progressive availability of EUS and the development of many types of needles made EUS-guided sampling a very important tool to advance personalized treatment through the sequencing of multiple genes. The molecular information obtained from EUS-guided tissue sampling plays a pivotal role in the identification of potential therapeutic targets for pancreatic cancer patients. This approach empowers oncologists to select personalized treatment regimens, address drug resistance, and explore innovative combination therapies, all aimed at improving patient outcomes and quality of life in the challenging landscape of pancreatic cancer. However, the difficulty in obtaining pancreatic tumor samples is still a major issue [30]; however, for the pathological diagnosis of a solid pancreatic mass, EUS tissue acquisition (EUS-TA) has extensively been proven to be highly accurate (sensitivity: 85–89%; specificity: 96–100%) by meta-analyses [31,32,33]. Being a safe, cost-effective, and accurate technique, EUS-TA has become the standard diagnostic test for PDAC. In the early 1990s, the first cases of endoscopic ultrasound-guided fine-needle aspiration (EUS-FNA) of pancreatic lesions using a curved linear-array echoendoscope were reported; subsequently, specifically designed needles for fine-needle biopsy (FNB) were created [34]. The difference between EUS-FNA and EUS-FNB is basically the structure of the needles. Thus, they are both categorized as EUS-TA.

Due to the evolution of needles, including the first-generation tru-cut-based biopsy technique (Quick-Core, Wilson-Cook, Winston-Salem, NC, USA), second-generation needles with whole-side bevels (ProCore, Wilson-Cook), and third-generation needles, there has been progressive improvement in terms of amounts of tissue with fewer needle passes to obtain adequate samples for diagnosis. Third-generation needles with Franseen-type bevels (Acquire, Boston Scientific, City, MA, USA) or fork-tip-type bevels (SharkCore, Medtronic, Dub, Ireland), which obtain large amounts of core tissue, are an important tool for immunostaining, molecular, and genetic analyses [35,36,37].

Although NGS can sequence multiple genes in limited samples [38,39,40], the acquisition of a relatively large sample is mandatory for NGS. The first topic is whether FNB is better than FNA. In terms of specimen adequacy for genomic profiling and DNA yield, Kandel et al. conducted a randomized trial including 50 patients with pancreatic masses who consecutively underwent both conventional EUS-FNA with a 25-gauge needle and EUS-FNB (19- or 22-gauge needle). The mean DNA concentrations in PDAC tissue obtained with FNB and FNA needles were, respectively, 5.930 (SD 0.881) µg/mL vs. 3.365 (SD 0.788) µg/mL (*p* = 0.01). Kandel et al. concluded that specimen adequacy for genomic profiling and DNA yield was significantly higher with FNB than FNA needles [41]. In another study by Park et al., factors associated with endoscopic ultrasound sampling for successful NGS in 190 patients with PDAC were investigated [28]. In univariate analysis, a larger needle gauge and tumor located in the body/tail were associated with successful NGS. In multivariate regression analysis, the gauge of the needle was confirmed to be the only significant factor for successful NGS. Interestingly, DNA amount, according to the EUS sampling needle type, was not significantly different between EUS-FNA and EUS-FNB. On the other hand, the DNA amount obtained with 25-gauge needles was significantly smaller than that obtained with 19- or 22-gauge needles. Moreover, DNA amount was significantly higher in the NGS success group than in the group who failed NGS (1.42 µg vs. 0.54 µg), thus concluding that the number of cells themselves may be a more important factor for successful NGS than the cellularity of the tumor. In a retrospective study, Ishigaki et al. found that EUS-TA, using a 22-gauge Franseen needle, enabled comprehensive genomic profiling (CGP) in solid tumors—78% of which were PDAC—with success rates of 86% for pathology review and 76% for the final CGP test. However, many samples did not meet ideal tissue/tumor content criteria, without significantly affecting outcomes [42].

A major issue in the genomic sequencing of PDAC is the small number of malignant epithelial cells of the cancer itself, which can negatively impact the sensitivity of mutation detection. PDAC is notably characterized by low neoplastic cellularity (5% to 20%) and a prominent desmoplastic reaction, invalidating samples’ adequacies [43]. Cellularity is an important factor for NGS, and tumor fraction > 20% is required [44]. Samples with low tumor fractions have an increased risk of false-negative results, so that the acquisition of large tumor samples to avoid contamination is mandatory for NGS [45]. To overcome NGS obstacles due to desmoplastic fibrosis, some studies propose that liver metastasis or lymph node metastasis could be used for EUS-guided tissue acquisition as a complementary approach for NGS [46].

Third-generation needles may enable the acquisition of adequate core tissue samples that can be processed in formalin-fixed paraffin-embedded (FFPE) specimens. FFPE specimens are still preferred for genomic profiling, since they provide direct evaluation of the tumor cellularity, and pathologists can easily select areas with higher cellularity in the samples for NGS. Furthermore, FFPE samples allow the preservation of tissue architecture allowing future ancillary studies [47]. Nevertheless, the quality of DNA in FFPE samples can be altered due to formalin fixation and processing [48]. Hartley et al. compared 30 matched EUS-FNA cytological smears and macro-dissected FFPE samples from surgical resection. The DNA yield per nuclear area was higher in the cytological smear compared to in FFPE samples (0.86 ng/mL vs. 0.51 ng/mL, respectively, *p* = 0.0051) [49].

Several studies have examined the surrogacy of EUS-TA samples of surgically resected specimens in NGS; finally, it was shown that high concordance exists between the results of NGS using both EUS-TA samples or a surgically resected specimen used as a reference standard [45]. In this context, Gleeson et al. showed the feasibility and ability to identify potential actionable mutations of EUS-FNA samples, with an NGS panel concordance in 83% of the cases between EUS-FNA samples and surgical resected specimens [50].

The adequacy of EUS-TA samples for NGS has been evaluated in several retrospective studies, which reported that the adequacy of EUS-TA samples for NGS ranged from 60% to 100%. In 2010, Fujita et al. reported a successful molecular analysis from fixed formalin specimens of PDAC collected by EUS-FNA [51]. Later, Larson et al., evaluating the adequacy of biopsy samples of 76 patients, reported an NGS success rate of 70.4% using EUS-FNB (54 patients) compared to the 42.9% of the EUS-FNA group [52]. Similarly, in 2020, Elhanafi et al. evaluated the adequacy of EUS samples in 167 patients with PDAC (145 EUS-FNA, 22 EUS-FNB). In this study, EUS-FNB with a 22-gauge needle had a higher proportion of adequate samples for genomic analysis compared with EUS-FNA (90.9% vs. 66.9%, respectively; *p* = 0.02) [53]. A retrospective study published in 2025, involving 86 patients with pancreatic cancer, reported a 66% success rate on the FoundationOne CDx assay when using EUS-guided tissue acquisition. Notably, the longer macroscopic core length was independently correlated with successful genomic profiling, emphasizing the relevance of obtaining adequate tissue samples [54].

In 2023, a randomized trial was conducted by Bang and colleagues to determine the optimal technique with which adequate DNA and RNA can be procured for comprehensive molecular profiling (CMP) studies [55]. Patients with pancreatic mass proven to have adenocarcinoma by rapid onsite evaluation at EUS were randomized intra- procedurally to undergo two (17 patients) or three (16 patients) FNB passes for CMP with 22-gauge Franseen needles. In both groups, all cases resulted in adequate DNA extraction, and no differences were observed in DNA concentration; thus, a specific needle gauge and a procedural maneuver are proposed for determining the finite number of biopsies required for the reliable procurement of tissue for CMP. More recently, a study investigated the feasibility of obtaining adequate DNA for molecular analysis from 43 PDAC EUS-FNB formalin-fixed paraffin-embedded (FFPE) specimens [56]. The authors verified the DNA adequacy in terms of quantity and quality and evaluated the MSI-H status by droplet digital PCR (ddPCR). The ddPCR identified MSI-H phenotype in 16.28% of cases, suggesting the possibility of improving the selection of patients who may benefit from immunotherapy.

Pancreatic cancer remains a formidable challenge in oncology, but advancements in personalized treatment approaches, particularly using endoscopic ultrasound (EUS) tissue sampling, have offered new hope. EUS, combined with molecular profiling techniques like next-generation sequencing (NGS), is revolutionizing the way we understand and treat this disease. The molecular information obtained from EUS-guided tissue sampling helps identify potential therapeutic targets.

## 4. Is DNA Enough?

Several studies have demonstrated the RNA role in cancer development, progression, and metastatic spread. Most RNA molecules do not take part in protein translation and are therefore termed “non-coding” RNAs; this family accounts for 95% of the RNA pool. Genome-wide association studies (GWASs) have highlighted the importance of DNA sequences containing non-coding RNAs (ncRNAs), which are associated with a variety of human diseases, including PDAC [57,58]. This is a heterogeneous group including “short” molecules such as microRNAs (miRNAs) and small interfering RNAs; “long” molecules are termed long non-coding RNAs (lncRNAs), including circular RNAs (circRNAs). miRNAs are short non-coding RNA molecules with a length of ~22 nt that regulate gene expression by binding to the 3′ untranslated region (UTR) of target mRNAs, leading to mRNA degradation or translational repression [59,60]. microRNA expressions are related to the type of tumor, as well as its occurrence and development. As miRNA binds mRNA through incomplete pairing, inhibiting translation, the same miRNA can also regulate several target genes and signaling pathways. miRNAs are also conservative and testable and can exist stably outside cells. For these reasons, they have become diagnostic markers and therapeutic targets of tumors. miRNAs can act as oncogenes (oncomiR) or tumor suppressor genes (tsmiRNA) in PDAC. Among these, mir-195, mir-190, mir-186, mir-221, mir-222, mir-200b, mir-15b, mir 25, and mir-95 were significantly upregulated, while let-7, mir-96, mir-15, and mir-132 were significantly downregulated. miR-25, miR-155, and miR-221/222 promote oncogenic processes, amongst which microRNA-25 is found to be significantly upregulated in pancreatic cancer patients, and there is an ongoing study for a potential diagnostic marker in PDAC [61,62]. Another study defined the role of miRNA 195 in normal pancreatic cells, pancreatic duct epithelial neoplasm (PanIN, precancerous) cells, and pancreatic cancer cells. It was found that, in normal pancreatic cells, mir-195 expression was not present; however, PanIN and all tumor cells showed positive expression of miR-195, suggesting that mir-195 might indicate the carcinogenic transformation process of pancreatic cells [63]. Quite the opposite, examples of miRNAs with better survival rates are the upregulated miR-142 and miR-204, while downregulated miR-19a-3p corresponds to higher survival impact [60,61].

Whilst long non-coding RNA sequences (lncRNA) are defined as RNA transcripts longer than 200 nucleotides with no protein-coding potential [64], LncRNA plays an essential role in the regulation of gene expression (epigenetic, transcriptional, or post-transcriptional) through interaction with other regulatory molecules such as microRNA or through direct binding to protein complexes such as transcriptor factors [65]. In LncRNA, there are single-nucleotide polymorphisms (SNPs), which could influence LncRNA expression and impact their function as regulators. At least three PDAC risk loci were identified by GWAS and have been reported to be in lncRNAs (rs6971499 at 7q32.3) (LINC-PINT), rs13303010 at 1p36.33 (NOC2L), and LINC00673-rs11655237. Another lncRNA rs7046076 in the region of SMC2, was reported to be suggestively associated with PDAC risk [66].

Among ncRNAs, there are circRNAs. circRNAs are covalently closed, single-stranded RNA molecules that form a covalently closed continuous loop structure. Emerging evidence suggests that these molecules play critical roles in pancreatic cancer development and progression by acting as miRNA sponges, modulating gene expression, and interacting with RNA-binding proteins [60,61]. For example, ciRS-7 (Cdr1as) is able to promote the EGFR/STAT3 pathway through the suppression of miR-7 in the ceRNA network and is correlated with increased cancer-associated endothelial cells coupled with pathological angiogenesis. Within PDAC, the overexpression of Circ-ASH2L has been proposed to stimulate angiogenesis through the upregulation of the Notch 1 pathway, in turn stimulating downstream VEGFa activity [67]. circRNAs are also involved in chemotherapy resistance: the expression profile of these molecules between PDAC cell lines, with and without gemcitabine resistance, has been shown to differ significantly. Furthermore, this dysregulation was shown to persist when comparing plasma samples from patients and was able to predict clinical response to gemcitabine compared to non-responders. Cells overexpressing ciRS-7 (CDR1as) are thought to demonstrate increased gemcitabine resistance because of impaired regulation of EGFR/STAT3 signaling pathway [67].

In this setting, in recent years, transcriptomic signatures have become promising tools for selected groups of genes. This analysis is a good indicator of the biological behavior of a tumor, as the quantification of RNA is the most comprehensive approach for determining the phenotype of a tissue.

Transcriptomic signatures have also been studied to select the most effective chemotherapy regimen in PDAC. Two main molecular subtypes of this cancer have been identified: classical PDAC characterized by better prognosis and response to Folfirinox and basal-like PDAC with a worse prognosis and a possible response to gemcitabine-based therapy [16,68]. From this perspective, an RNA signature from basal-like PDAC containing thousands of transcripts was studied. The presence of this RNA signature was a positive predictor of OS and PFS in gemcitabine-treated patients [69].

Single-cell RNA sequencing (scRNA-seq) has recently emerged as a valuable tool in precision medicine by enabling in-depth characterization of tumor cell and tumor microenvironment also with low amounts of tissue. The PDAC tumor microenvironment (TME) is a critical issue of therapeutic resistance and tumor progression. A recent study, through single-cell RNA sequencing, highlights the effect of chemotherapy on PDAC TME: both Folfirinox and gemcitabine/abraxane deeply alter the tumor microenvironment and might lead to further resistance to immunotherapy by reduced inhibitory checkpoint molecule expression and interactions involving CD8 +  T cells [70].

However, RNA is difficult to extract from a pancreatic tumor because of the abundance of RNAse in the pancreatic gland, which leads to fast RNA degradation, and because of the abundance of stroma compared to tumoral cells. Many studies have been conducted in this sense, but based on surgical samples, only a few reports exist on RNA extraction through EUS-TA (Table 2). Berry et al. reported an amount of 12.9 ug of low-quality RNA retrieved using one pass of FNA frozen in liquid nitrogen [71]. Another study demonstrated that only 28.8% of archival FFPE EUS-FNB samples resulted in adequate material for nanostring analysis [72]. Recently, Archibugi et al. investigated the best methodology for RNA extraction from EUS-TA and the feasibility of transcriptome analysis including the PDAC molecular subtype and splice variant expression: EUS-acquired samples of PDAC conserved in Trizol resulted in a significantly higher concentration of RNA, suitable for conducting qPCR for tissue and prognostic biomarkers such as GATA6 and ZEB1 and for determining splicing events [73].

## 5. Conclusions

In recent years, the development of molecular genomics assays to detect genomic biomarkers has improved the concept of precision medicine to treat patients with PDAC. Endoscopic ultrasound, fine-needle biopsy, which can reliably collect tissue from pancreas, has the potential to improve personalized treatment in pancreatic tumor by allowing the assessment of genomic alterations and eventually leading to a better prognosis and a greater survival of patients soon. Personalized treatment approaches in pancreatic cancer, guided by EUS-guided tissue sampling and molecular profiling techniques, represent a promising frontier in oncology. By tailoring therapies to the unique genetic makeup of each patient’s tumor, clinicians aim to maximize treatment effectiveness, minimize side effects, and ultimately improve the prognosis and quality of life for individuals facing this challenging disease. The integration of EUS into the personalized treatment paradigm is a testament to the progress being made in the field of pancreatic cancer research and care. The continuous discovery of novel genetic alterations through EUS-guided tissue sampling fuels the development of targeted agents. Pharmaceutical companies are actively working on drugs that can specifically target various genetic alterations found in pancreatic cancer. Future clinical trials are essential for evaluating the safety and efficacy of new treatments in pancreatic cancer. To improve patient outcomes and safety, researchers are increasingly relying on molecular data obtained from EUS-guided tissue sampling to design clinical trials that target specific genetic alterations. This approach holds great promise for developing more effective therapies tailored to the unique molecular profiles of pancreatic cancer patients. This ongoing research expands the repertoire of targeted therapies available for patients. In summary, the molecular information obtained from EUS-guided tissue sampling plays a pivotal role in the identification of potential therapeutic targets for pancreatic cancer patients. This approach empowers oncologists to select personalized treatment regimens, address drug resistance, and explore innovative combination therapies, all aimed at improving patient outcomes and quality of life in the challenging landscape of pancreatic can.

## Figures and Tables

**Table 1:** Summary table of major studies.

Study	Design and Settings	No. of Patients	NGS Success Rate	Actionable Mutations (%)	Clinical Impact Summary
COMPASS	Prospective, multicenter (Canada), RNA and DNA sequencing in metastatic PDAC	63 patients with biopsy	98% for whole-genome sequencing and 95 for RNA sequecing	32.3%	Identified molecular subtypes and potential treatment responses. Basal-like subtype had shorter median treatment duration (1.5 months) compared to classical subtype (4 months).
Precision-Pan**c**	UK-based umbrella trial with multiple arms and molecular subtyping—ongoing	Data not specified	Data not specified	Data not specified	Aimed to match patients to trials based on molecular profiles, enhancing personalized treatment approaches.
Know Your Tumor	Retrospective, multicenter (USA), targeted NGS in PDAC patients	640 patients	96%	50% (highly actionable and modified options)	At least 1 pathogenic mutation in 616 patients, with a median of 4 per patient. Most common mutations were in the MAPK pathway, predominantly KRAS. Identified actionable mutations in DNA repair genes, mostly ATM and BRCA2.
IMPaCT	Prospective, multicenter (Australia), NGS for recurrent/metastatic PDAC	93 patients considered; 76 patients processed for NGS-74 NGS available	97.4%	22 patients with genetic target (30%)	No patient has been successfully treated with targeted therapies.

**Table 2:** Summary table of studies addressing RNA extraction from EUS-TA.

Study	Needles Type	Preservation Method	RNA Quality/Integrity	Success Rate	Feasibility for Transcriptomic Analysis	Remarks
Berry et al. (2017) [71]	22G EUS-FNA	Snap freezing (immediate storage in liquid nitrogen at −80°).	RNA quality sufficient for qPCR and xenograft model development (mean RIN 3)	Not explicitly stated	Partial—used for gene expression of KRAS status	Focused on generating patient-derived xenograft (PDX) models; limited transcriptomic profiling
Gleeson et al. (2020) [72]	22G EUS-FNB	FFPE	Not RIN-based; quality verified via Nanostring platform (no amplification needed)	High (all samples yielded usable gene signatures)	Yes—digital mRNA profiling more than 730 immune-related genes using Nanostring nCounter^®^	Demonstrated feasibility of immunophenotyping PDAC from FFPE EUS-FNB samples—target for immunotherapy
Archibugi et al. (2020) [73]	25G FNA, 20G lateral-core FNB, 25G FNB	The first set was positioned in dry ice and then stored at −80 °C. The second set was stored in a vial containing 600 uL of RNALater and then stored at −80 °C. The third set was positioned in dry ice, 1000 uL of Trizol was then added, and then the set was stored at −80 °C.	In Trizol: Median RIN: 5.15; RNA concentration: ~10.33 ng/µL In dry ice (snap frozen): Median RIN: 5.85; RNA concentration: ~0.64 ng/µL	~75% success for qRT-PCR and transcriptomic profiling in Trizol	Yes—used for NanoString PanCancer panels and KRAS mutation analysis	EUS-acquired samples of PDAC conserved in Trizol resulted in a significantly higher concentration and sufficient integrity of RNA and is hence usable to perform qPCR for tissue and prognostic biomarkers, as well as the evaluation of splicing events
